# Indoor positioning with multi-domain CSI-based deep attention networks for MIMO wireless systems

**DOI:** 10.1038/s44459-025-00021-y

**Published:** 2026-05-04

**Authors:** Praneeth Susarla, Anirban Mukherjee, S. S. Krishna Chaitanya Bulusu, Pravallika Katragunta, Dinesh Babu Jayagopi, Miguel Bordallo López, Markku Juntti

**Affiliations:** 1 Centre for Wireless Communications, University of Oulu, Oulu, Finland; 2 Multimodal Perception Lab, IIIT Bangalore, Electronic city, Karnataka India; 3 École Centrale School of Engineering, Mahindra University, Hyderabad, India; 4 Department of Computer Science, Carleton University, Ottawa ON, Canada; 5 Center for Machine Vision and Signal Analysis, University of Oulu, Oulu, Finland

**Keywords:** Information technology, Mathematics and computing

## Abstract

Accurate indoor positioning is vital for applications such as augmented reality and autonomous robotics. Channel state information (CSI)-based methods, particularly when combined with beamforming, massive multiple input multiple output (mMIMO) techniques, and artificial intelligence (AI) algorithms, offer enhanced indoor user equipment (UE) positioning accuracy and robustness in complex indoor environments. In this paper, we present an AI-driven CSI-based indoor positioning method for mMIMO systems, where channel impulse, channel frequency, and angular response domain features are extracted from the CSI data and combined to form both uni-domain and multi-domain feature sets. We introduce a deep attention network (DAN), an AI algorithm that leverages attention mechanisms to effectively integrate and process multi-domain CSI data, thereby enhancing UE positioning performance. We evaluate DAN using a publicly available mMIMO dataset and compare its performance against the baseline and multi-domain convolutional neural network (CNN) models. Our results show that multi-domain DAN outperforms CNN approaches in positioning accuracy, though at the cost of increased inference complexity-highlighting a trade-off between performance and computational overhead. These findings demonstrate the potential of attention mechanisms and multi-domain CSI features for accurate indoor UE positioning systems.

## Introduction

Indoor user equipment (UE) positioning is emerging as the key enabler for applications, such as connected robots, digital twins, augmented reality, etc [1, 2]. It has been extensively investigated over the recent decades, especially in industrial settings and for wireless sensor networks [3]. Global Navigation Satellite Systems (GNSS) often struggle to accurately pinpoint users in indoor environments [4, 5]. A growing trend to address this challenge is to reduce system costs by using existing wireless infrastructure as reference points for indoor positioning systems [6]. This has resulted in a growing interest in parameter-based algorithms for in-building positioning in recent years, with some incorporating machine learning techniques. Wireless measurements with parameters, such as channel state information (CSI), received signal strength indicator (RSSI), and angle of arrival (AoA) are influenced by human movements, with CSI RSSI being range-based parameters and AoA being angle-based parameters [7]. These measurements, obtained from communications between sixth generation (6G) massive multiple input multiple output (mMIMO) wireless systems, can also be used for human positioning and sensing without adding additional overhead to the communications environment [6, 8]. CSI-based methods, particularly when combined with beamforming, large antenna arrays, and multiple input multiple output (MIMO) techniques from fifth generation (5G) and 6G wireless systems, can allow for more precise sensing, with very accurate measurements of distance, speed, and direction, as well as location tracking down to centimeter-level accuracy [9].

This paper addresses indoor positioning to estimate the location of a user or device in an indoor environment. We confine to CSI in this work, as it has attracted many research efforts and some pioneering works have demonstrated sub-meter or even centimeter-level accuracy in indoor positioning [10]. CSI describes the characteristics of a wireless communication channel, including factors, such as signal strength, channel propagation, and interference. It helps to optimize wireless communications between IoT devices and networks by adjusting transmission parameters for improved reliability. CSI also enables advanced techniques like beamforming and MIMO to enhance communications performance. CSI contains spatial information that base stations (BSs) uses to multiplex users in the spatial domain, which can be extracted and utilized for user positioning. The recent research in [11–22] emphasizes the usage of neural networks (NN) for CSI-based positioning in indoor environments. Among them, convolutional neural networks (CNNs) have been the most commonly used NN architecture in the recent literature [11–15]. The authors in [11] perform CSI-based indoor positioning by constructing CNN images using estimated AoA values from received packets at the access point. In [12], authors deploy the CNNs architecture over the extracted features from CSI information, obtained from a single mMIMO antenna. Here, the extracted features essentially contain the polar and raw CSI features from the channel frequency response (CFR) domain and the Cartesian form of the channel impulse response (CIR)-domain information from CSI information. The authors in [13] improve the CNN architecture by incorporating residual network (ResNet) blocks and applying them over the same CSI feature data. In [14, 15], authors perform indoor positioning by building spatio-temporal features over CSI amplitude and phase information and design their respective CNNs.

In recent times, different NN architectures, such as feedforward NN [16, 17], transformers [18], federated learning [19, 20], etc. are also employed for indoor positioning over 5G and cellular-based CSI information. Also, CSI-based positioning has been performed using NN-based fusion architectures [16, 20] and ensemble NNs [21]. We observe that carefully designed features from CSI information are critical for all of these methods, as real-world channels exhibit small-scale fading and wireless transceivers suffer from several hardware impairments. All the above-mentioned works, extract various features from raw CSI data, such as amplitude, phase, polar-form of features, and auto-correlation, across different domains including CFR or CIR.

In our recent work in [23], we explored the fusion of CSI-extracted features from multiple domains, such as CFR, CIR, and channel angular response (CAR), and demonstrated that such multi-domain representations enhance positioning performance. Building upon this foundation, this paper presents a unified multi-domain learning framework that systematically integrates frequency-, antenna spacing-, and angular-domain CSI representations through attention-based feature fusion. We design the deep attention networks (DAN) architectures, such as self-attention and cross-attention for CSI-based indoor positioning. Unlike prior works that employed individual domain features or heuristic fusion of multiple NN, our proposed framework adaptively learns interdependencies among CFR, CIR, and CAR domains within a single end-to-end model, enabling improved generalization across diverse system configurations. We analyze the combination of multi-domain feature selection extracted from CSI data using the designed deep attention architectures. We use the publicly available mMIMO CSI dataset (https://ieee-dataport.org/open-access/ultra-dense-indoor-mamimo-csi-dataset) to deploy our proposed architectures and compare their performance against the dataset CNN baseline methodology [12]. Furthermore, this paper extends our previous work [23] by providing a deeper investigation of the multi-domain DAN framework, emphasizing the relevance and interaction of multi-domain CSI features. We also conduct a comprehensive experimental evaluation across different feature sets, training subsets, attention mechanisms, and antenna configurations. The results show that the proposed multi-domain DAN framework achieves consistent and reliable positioning performance, making it well-suited for sub-10 cm indoor localization applications, such as industrial automation, AR/VR, and robotic navigation. The main contributions of this paper are summarized as follows:

- We systematically investigate the CAR, CFR, and CIR domain representations for CSI-based indoor positioning and demonstrate that jointly learning multi-domain CSI features captures complementary spatial cues, thereby significantly enhancing positioning accuracy.
- We perform the computational complexity analysis of individual multi-domain CSI features and the defined feature sets, measured in terms of floating point operations (FLOPs). This quantifies the trade-off between the accuracy gains and computational efficiency achieved through multi-domain fusion.
- We design the multi-domain DAN framework using CNN and attention blocks to perform CSI-based indoor positioning effectively across different antenna configurations. The proposed attention-based fusion mechanism enables the model to emphasize informative domain components and suppress redundant ones, improving position inference accuracy.
- We analyze the performance of the multi-domain DAN framework under varying sizes of CSI training datasets. We observe that while CNN performs better with extremely limited data, DAN achieves up to 40% lower mean positioning error once the training data exceeds 5%, demonstrating its superior scalability and data efficiency.
- We compare the inference complexities of the proposed multi-domain DAN with multi-domain CNN benchmarks. The results show that although the DAN architecture incurs a higher inference time due to attention operations, it consistently achieves sub-10 cm positioning accuracy, outperforming CNN models trained on comparable datasets.

The rest of the paper is organized as follows: "Results" section presents our proposed multi-domain DAN and CNN baseline approaches and compares them against the CNN benchmark results. Discussion section summarizes the conclusion and future work. Methods section describes the indoor mMIMO CSI dataset setup, feature sets’ construction and their complexity, CNN baseline, and the proposed DAN architectures.

## Results

In this section, we implement the CNN and DAN architectures described in section “CNN Baseline architecture for Multi-Domain” and section “Multi-domain Deep Attention Network”, respectively. We obtain the CSI information from the indoor mMIMO dataset [24] and construct the Cartesian, polar features for CIR, CFR, and CAR domains as described in section “Multi-domain CSI Feature Set Construction”. We construct these features using CFR information from two curated public subsets, UltraDense-15K and UltraDense-50K, derived from the publicly available mMIMO dataset, supporting reproducibility.

We consider the CNN-based indoor CSI positioning in [12], as the benchmark and evaluate the multi-domain feature performance over CNN baseline and proposed DAN architectures. We first investigate the performance of CNN-based positioning across various combinations of these domains, using multi-domain feature sets defined in Table 4. Following this, we compare the performance of the proposed multi-domain DAN approach against the multi-domain CNN baseline approach and also CNN benchmark results from [12]. Next, we assess the performance of multi-domain DAN across different antenna configurations, multiple labeled datasets, as well as different sizes of the training for the selected UltraDense-15K labeled dataset. Lastly, we also compare the multi-domain inference complexities of DAN and CNN architectures relative to the CNN benchmark inference complexity in [12]. All these simulations are implemented using Python and the GitHub link is provided in [25]. We define the metric mean absolute error (MAE) on the test dataset for each experiment with a feature set as follows:

$$\,{\mathrm{MAE}}\,={\mathbb{E}}\left\{| p-\hat{p}| \right\},$$

where *p* is the measured position of the user, i.e. ground truth and $\hat{p}$ is the estimated position and position error is ${\boldsymbol{X}}=| p-\hat{p}|$. Note that we used mean square error ${\mathbb{E}}\left\{| p-\hat{p}{| }^{2}\right\}$ as the cost function when training the models. However, for a fair comparison of our results with the benchmark results in [12] we use MAE on the test dataset. We use kernel density estimate (KDE) (analogous to histogram) to analyze MAE distribution of CSI-based positioning errors, using a continuous probability density curve in one or more dimensions. KDE provides a smooth, non-parametric estimate of the probability density function PDF for the observed positioning errors across all test samples. It is a way to smooth out a histogram to a more continuous and informative representation of the data’s distribution [26]. Given a set of position error observations {*x*_1_, *x*_2_, . . . , *x*_*n*_} drawn from an unknown distribution, KDE at a point *x* is defined as

$${\hat{f}}_{X}(x)=\frac{1}{nh}\mathop{\sum }\limits_{i=1}^{n}{\mathcal{K}}\left(\frac{x-{x}_{i}}{h}\right),$$

where *n* is the number of data points, *x* is the point at which the density is estimated, *x*_*i*_ is the *i*th data point, ${\mathcal{K}}(.)$ is the kernel function (Gaussian kernel is most popular due to its smoothness), a symmetric and non-negative function that integrates to 1, and *h* is the bandwidth or smoothing parameter, which controls the width of the kernel and thus the smoothness of the estimate. Note that the small *h* implies overfitting (noisy, wiggly curve) and large *h* implies underfitting (oversmoothed, loses details).

By plotting the KDE with respect to positioning error, we visualize the frequency of different error magnitudes in localization. The KDE curve reflects the empirical likelihood of achieving a particular level of accuracy; its peak (mode) indicates the error value that occurs most often, while the tails represent rarer, larger errors.

Specifically, as positioning error increases from zero, the KDE curve starts low, rises to a peak, and then decreases. This pattern means that most localization results are concentrated near the mode, with both extremely small and large errors being less common. Interpreting the KDE curve allows us to assess not only average accuracy but also the consistency and robustness of the proposed methods, as well as the risk of encountering outlier errors. Thus, the KDE vs. positioning error plot provides a comprehensive summary of the practical error distribution and is more informative than simple summary statistics like mean or median error. A summary of the metrics obtained from CNN and DAN approaches, using the kernel density estimates of UE position prediction error ***x*** on the test dataset are listed in Table 1.

**Table 1:** Statistical summary of UE position prediction error (*x*), including mean absolute error (MAE), MAE in wavelengths (*λ*), and mode (μ), derived from the kernel density estimates *f*_*X*_(*x*) on the mMIMO UltraDense-15K test dataset for CNN, DAN, benchmark CNN without TL, and benchmark CNN with TL approaches with multiple feature set representations

Model	Feature set^*a*^	Positioning Error^*b*^		
		MAE (mm)	Mode *μ* (mm)	MAE (*λ*)
CNN	U_1_	39.20	25.21	0.34
CNN	U_2_	34.76	25.22	0.30
CNN	U_3_	34.44	21.49	0.30
CNN	M_1_	32.10	23.94	0.28
CNN	M_2_	31.70	22.26	0.28
CNN	M_3_	35.61	22.98	0.31
CNN	M_4_	30.52	19.20	0.27
Benchmark CNN without TL [12]	M_b_	59.05	-	0.51
Benchmark CNN with TL and 10k samples [12]	M_b_	42.79	-	0.37
**Proposed DAN**	M_4_	**30.09**	**17.44**	**0.26**

^*a*^Here, U_1_, U_2_, U_3_, M_1_, M_2_, M_3_, M_4_, and M_*b*_ are the feature sets defined in Table 4.

^*b*^The results are presented in absolute and relative accuracies using millimeters and wavelength (i.e., *λ*) as the units, respectively.

### Unidomain Comparison

This section highlights the analysis observed from CNN baseline for U_1_, U_2_, and U_3_ uni-domain feature sets defined in Table 4. A detailed analysis of this study is already presented in our recent work [23]. Although the Cartesian and polar forms contain equivalent CSI information in every domain, they emphasize different channel characteristics. The Cartesian form captures spatial and interference-related variations through real and imaginary components, whereas the polar form separates magnitude and phase, revealing power and propagation geometry. Since standard NN are real-valued, combining both forms provides complementary feature representations that improve feature diversity and discriminability. In our previous work, we observed that this joint representation enables CNNs and attention mechanisms to better exploit spatial-spectral correlations, thereby enhancing position estimation performance. We also observed that capturing UE beam space or CAR information across multiple subcarrier frequencies from CFR measurements improves indoor positioning accuracies using CNN.

### Multi-domain CNN Baseline

**Fig. 1:**
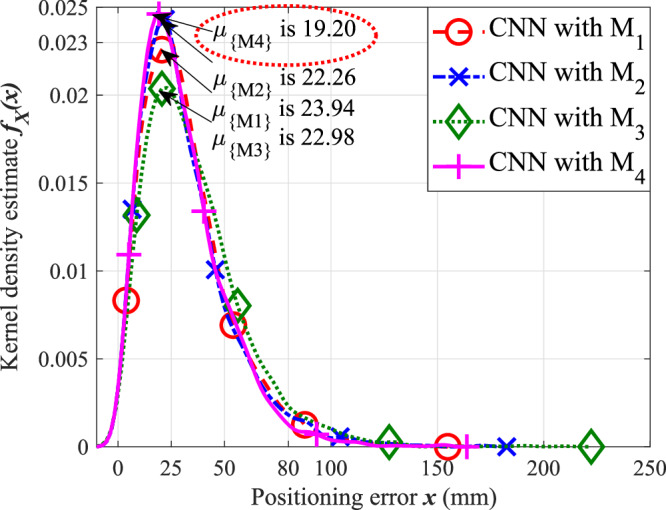
Comparison of density estimates of positioning error (*x*) when trained with multi-domain feature sets under the ULA scenario. The figure illustrates the performance distribution of the proposed and baseline methods, highlighting the improved consistency and reduced positioning error variance achieved by multi-domain feature learning.

This section highlights the analysis observed from CNN baseline for M_1_, M_2_, M_3_, and M_4_ multi-domain feature sets defined in Table 4. Red, blue, green, and magenta plots in Fig. 1 represent the KDE *f*_*X*_(*x*) of predicted UE positioning error on the test dataset when CNN baseline is trained with M_1_, M_2_, M_3_, and M_4_ feature sets, respectively.

Using the same CNN baseline architecture, we observe that multi-domain feature combinations lead to improved position prediction performance with lower mode compared to uni-domain feature set position prediction accuracies in [23]. Fig. 1 indicates that mode reduces by 1.68 mm when the CNN baseline is trained with the M_2_ (CFR and CAR) feature set compared to the M_1_ (CIR and CFR) set, highlighting the value of spatial spectrum (CAR) features for enhancing estimation performance. In contrast, we observe that mode increases by 0.72 mm when using the M_3_ (CIR and CAR) set compared to the M_2_ set, thereby emphasizing that CFR domain features must be included for accurate positioning. Notably, we observe that the M_4_ feature set yields the best reduction in position estimation error (*μ*_{*M*4}_ = 19.20 mm), indicating that incorporating CIR, CFR, and CAR domain features trains CNN models with the highest positioning accuracy.

### Multi-Domain CNN vs Multi-Domain DAN vs Benchmark Comparison

**Fig. 2:**
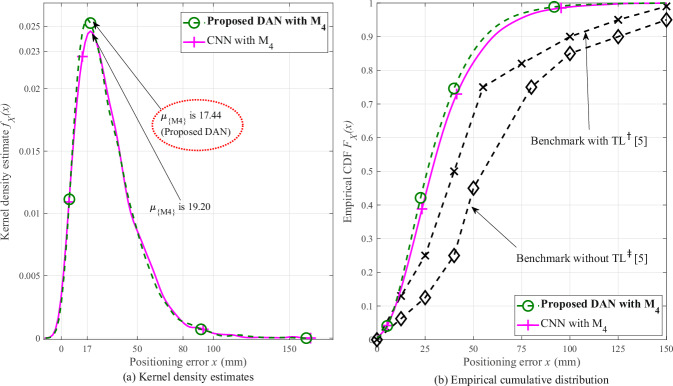
Performance comparison of the proposed DAN and CNN baseline approaches on the UltraDense-15K dataset using the M4 feature set. **(a)** Comparison of kernel density estimates of positioning error (***x***) for the CNN baseline and the proposed DAN approach under the ULA scenario. **b** Empirical cumulative distribution function (CDF) *F*_*X*_(*x*) of positioning error (***x***) for the CNN baseline with M_4_, the proposed DAN with M_4_, the benchmark with TL, and the benchmark without TL models. The proposed CSI-based positioning approach introduced in this paper is highlighted in bold. The numerical comparison of these methods is provided in Table 2.

This section evaluates the performance comparison for CNN baseline with M_4_, DAN with M_4_ using UltraDense-15K dataset, against the CNN benchmark values in [12]. Green and magenta plots in Fig. 2a, Fig. 2b represent the KDE, cumulative distribution functionCDF estimates for UE position prediction error ***x*** using CNN baseline and DAN approaches with M_4_ feature set, respectively. From Fig. 2a, we observe that mode reduces by 1.76 mm when DAN is trained with M_4_ feature set, as opposed to CNN trained with the same feature set. Also from Table 1, we observe that DAN achieves the lowest mode of 17.44 mm (0.26*λ* in terms of wavelength) compared to all CNN models with various feature sets.

The black plots with ‘x’ and ‘♢’ markers in Fig. 2b represent the empirical CDF points for the CNN benchmark trained on 214,200 CSI samples (referred to as ‘benchmark without transfer learning (TL)’) and the CNN benchmark trained with TL on 10*k* CSI samples (referred to as ‘benchmark with TL’), respectively. Both sets of these points are referenced from [12]. Table 2 highlights the comparison of CNN baseline with M_4_ and DAN with M_4_ against the two benchmark results. From the cumulative probability column in the table, we observe that using DAN nearly half of the test dataset samples can be predicted within 25 mm positioning error. The same positioning error range can be achieved using the ‘benchmark with TL’ model only to a quarter of the test dataset samples. From the positioning error column in Table 2, we also observe that three-fourths of the test dataset samples can be predicted with a positioning error of 80 mm using ‘benchmark without TL’. The proposed DAN approach when trained with 15,000 CSI samples and M_4_ feature set, achieves the same with nearly halving (44 mm) the positioning error. Therefore, Table 2 shows that both CNN baseline with M_4_, proposed DAN with M_4_ approaches achieve higher position prediction accuracies compared to the benchmark results. Thus, multi-domain features and attention mechanisms can be useful (as evident from Table 2) to enhance indoor positioning accuracy from CSI-extracted information.

**Table 2:** Comparison of empirical cumulative distribution function (CDF) points corresponding to Fig. 2b for the UltraDense-15K test dataset under the ULA configuration

Model	*D*^*a*^	Cumulative probability	Positioning error
		*P*(*X* ≤ 25)	*x* at *P*(*X*) = 0.75
Benchmark without TL^*b*^[12]	214,200	0.13	80
Benchmark with TL^*c*^[12]	10,000	0.25	50
CNN baseline with M_4_	15,000	0.42	47
**Proposed DAN with** M_4_	**15,000**	**0.48**	**44**

The table summarizes the cumulative probability *P* (*X* ≤ 25) P(X ≤ 25) and the positioning error at *P* (*X*) = 0.75 P(X)=0.75 for proposed DAN with M4, CNN baseline with M4, benchmark without TL, and benchmark with TL models, demonstrating the improved performance of the proposed DAN approach with the *M*4 feature set compared to the CNN baseline and benchmark methods. The best performance model and its corresponding values are highlighted in bold.

^*a*^*D* denotes the training dataset size (number of CSI samples).

^*b*^CNN model trained using 85% of 252,004 CSI samples in the ULA scenario.

^*c*^CNN model trained with TL using 10,000 CSI samples in the ULA scenario.

### Multi-domain DAN performance across different antenna configurations, architectures, and UltraDense datasets

**Fig. 3:**
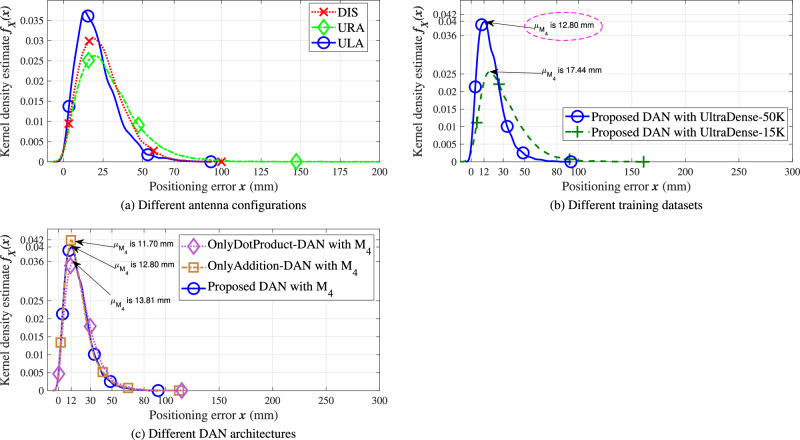
Performance of the proposed multi-domain DAN approach across different antenna configurations, UltraDense datasets and attention architectures. **a** Comparison of the KDE performance of the proposed DAN approach in inference mode across ULA, URA, and DIS antenna configurations. The blue, red, and green plots correspond to DAN models trained on the M_4_ feature set using the UltraDense-50K training dataset. **b** KDE performance comparison of the proposed DAN approach under the ULA-LoS configuration for the UltraDense-50K and UltraDense-15K datasets. **c** KDE performance comparison among the proposed DAN, OnlyAddition-DAN, and OnlyDotProduct-DAN architectures trained with the M_4_ feature set over the UltraDense-50K dataset.

This section evaluates the performance of the proposed DAN approach across different antenna configurations and UltraDense labeled datasets. Blue, green, and red plots in Fig. 3a illustrate the KDE estimates of the UE positioning error *x* for ULA, uniform rectangular array (URA), and distributed array (DIS) antenna configurations with line-of-sightLoS conditions, respectively. For each antenna configuration, the proposed DAN approach is trained on UltraDense-50K dataset with M_4_ feature set, and the trained model is used in the inference mode to compute KDE for UE positioning errors on the UltraDense-50K test dataset. From these plots, we observe that the KDE curves exhibit similar mode errors, indicating that the proposed DAN approach with the multi-domain M_4_ feature set achieves consistent performance regardless of the antenna configuration.

Blue and green plots in Fig. 3b illustrate the KDE performance of the proposed DAN approach under ULA-LoS configuration for UltraDense-50K and UltraDense-15K datasets, respectively. Note that we perform this simulation to understand the reliability of the proposed DAN approach over a large number of CSI samples. We observe that both plots achieve reliable positioning performance over the 15,000 and 50,000 CSI samples, further emphasizing the robustness of the proposed DAN approach. Furthermore, we also observe a slight reduction in mode error of 4 mm when using the larger UltraDense-50K data set. This suggests that increasing the training dataset size can improve the position prediction accuracies, albeit at the cost of significantly increased training time for the DAN models. Thus, while larger datasets may offer slight performance improvements, the trade-off with computational efficiency should be considered.

We further perform an ablation study to evaluate the individual contributions of the *additive* and *dot-product* attention mechanisms within the proposed DAN framework. Specifically, two additional variants are designed: *onlyAddition-DAN*, which integrates additive (Bahdanau-style) attention in all attention layers, and *onlyDotProduct-DAN*, which employs scaled dot-product attention throughout the network. Figure 3c shows the KDE plots of these two variants along with the hybrid DAN model on the UltraDense-50K test set. The *onlyAddition-DAN* achieves slightly higher positioning accuracy compared to the hybrid DAN and *onlyDotProduct-DAN* models when trained with the M_4_ feature set. However, as summarized in Table 3, it incurs a higher parameter count and longer inference time, while the *onlyDotProduct-DAN* exhibits lower latency at a modest cost in accuracy Table 4.

**Table 3:** Comparison of inference complexities for the CNN with M_4_, DAN with M_4_, and CNN with M_b_ models trained on the UltraDense-50K dataset

Model	Inference Weights	Hardware	Inference
		runtime	time^*a*^
	(Number of parameters)	(GFLOPs)	(seconds)
CNN with M_*b*_	3,233,058	0.078215394	26.82
CNN with M_4_	6,382,194	0.21972605	31.17
OnlyDotProduct-DAN with M_4_	6,380,146	0.22510512	37.5
**Proposed DAN with** M_4_	6,380,163	0.22665341	57.2
OnlyAdditive-DAN with M_4_	6,380,170	0.22671792	58.1

The table reports the number of inference weights (parameters), hardware runtime in GFLOPs, and inference time for 50,000 CSI samples, measured on a 5 GB NVIDIA A100 GPU.

^*a*^Inference time is measured in seconds for 50,000 CSI samples on a 5 GB NVIDIA A100 GPU.

**Table 4:** Feature sets constructed from multi-domain CSI features

Feature	Set	Domain			Form		Feature Set
Set	elements	CIR	CFR	CAR	Cartesian	Polar	Complexity^*a*^
U_1_	{**f**_1_, **f**_2_}		*✓*		*✓*	*✓*	6*NN*_*t*_
U_2_	{**f**_3_, **f**_4_}	*✓*			*✓*	*✓*	$6N{N}_{t}(1+{\log }_{2}N)$
U_3_	{**f**_5_, **f**_6_}			*✓*	*✓*	*✓*	$6N{N}_{t}(1+{\log }_{2}N)$
M_1_	{**f**_1_, **f**_2_, **f**_3_, **f**_4_}	*✓*	*✓*		*✓*	*✓*	$6N{N}_{t}(2+{\log }_{2}N)$
M_2_	{**f**_1_, **f**_2_, **f**_5_, **f**_6_}		*✓*	*✓*	*✓*	*✓*	$6N{N}_{t}(2+{\log }_{2}N)$
M_3_	{**f**_3_, **f**_4_, **f**_5_, **f**_6_}	*✓*		*✓*	*✓*	*✓*	$6N{N}_{t}(2+{\log }_{2}N)$
M_4_	{**f**_1_, **f**_2_, **f**_3_, **f**_4_, **f**_5_, **f**_6_}	*✓*	*✓*	*✓*	*✓*	*✓*	$6N{N}_{t}(3+{\log }_{2}N)$
M_b_^b^	{**f**_1_, **f**_2_, **f**_3_}	*✓*	*✓*		*✓*	*✓*	$6N{N}_{t}(1+{\log }_{2}N)$

Each feature set combines subsets derived from the CIR, CFR, and CAR domains in Cartesian and polar forms. The table also includes the computational complexity of each feature set, expressed in terms of FLOPs. M_b_ denotes the benchmark feature set defined in 12.

^*a*^ M_b_refers to the feature set used in the benchmark result in [12].

^*b*^Feature set complexity is measured in terms of floating-point operations (FLOPs).

Within the proposed DAN architecture, the additive attention mechanism captures complex feature relationships through a learned non-linear compatibility function, improving representational richness but increasing computational overhead. In contrast, the dot-product attention mechanism computes direct similarity between query and key vectors, offering parameter efficiency, full parallelizability, and reduced inference latency. By combining both mechanisms—additive attention in the deeper layers and dot-product attention in the higher layers—the proposed DAN achieves an effective balance between accuracy and computational efficiency.

### Multi-domain DAN performance over different sizes of the training dataset

**Fig. 4:**
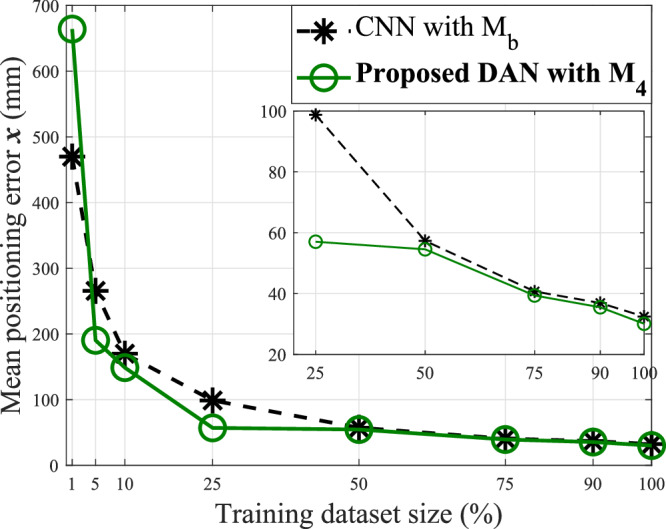
Performance comparison of mean positioning errors against different training dataset sizes for CNN baseline with M_b_ and the proposed DAN with M_4_ feature set observed over UltraDense-15K dataset. The results demonstrate the impact of training data volume on model accuracy and convergence behavior.

This section evaluates the performance of the proposed DAN approach across varying sizes of the training dataset. Figure 4 presents the mean UE positioning estimation error, *x*, on the UltraDense-15K test dataset, plotted against different percentages of the UltraDense-15K training dataset. The green curve represents the mean positioning error *x* using the proposed DAN approach with the M_4_ feature set, while the black curve corresponds to the CNN benchmark with the M_b_ feature set. A zoomed-in view of the plot highlights the mean positioning errors for both approaches when the training dataset size ranges from 25 to 100% of the UltraDense-15K training dataset. Here, 100% refers to the full UltraDense-15K training dataset, consisting of 12,000 CSI samples.

The CNN with M_b_ approach refers to the CNN architecture described in section CNN “Baseline architecture for Multi-Domain,” applied to the M_b_ feature set as outlined in [12]. Note that the ’benchmark without TL’ results are not suitable for comparison. Therefore, we implement a CNN with M_b_ as a reference to the approach proposed in [12]. We observe that the mean positioning errors on the UltraDense-15K test dataset increase as the number of CSI samples in the training dataset decreases, indicating that both deep learning architectures require sufficient training data with multi-domain features to achieve accurate positioning. Notably, the proposed multi-domain DAN with the M_4_ feature set outperforms the CNN architecture with M_b_, even when trained with a smaller number of CSI samples. This demonstrates that incorporating multi-domain feature sets as inputs significantly enhances positioning performance, even with limited training data. However, when only 1% of the training dataset (120 CSI samples) is used, the CNN performs better than the DAN. This highlights that attention-based methodologies like the DAN can be significantly impacted by very limited training samples, even when leveraging multi-domain features.

### Multi-domain CNN, DAN inference complexities and prediction

This section examines the inference complexities of multi-domain CNN and DAN models relative to the CNN benchmark after analyzing their performance on multi-domain CSI feature sets. The multi-domain CNN and DAN models correspond to the CNN baseline and the proposed DAN architectures, respectively, both trained using the M_4_ feature set and UltraDense-50K dataset. In contrast, the CNN benchmark represents the CNN baseline architecture without any added fully connected layer and trained with the M_b_ feature set as outlined in [12]. Table 3 provides a detailed comparison of inference complexities for the CNN with M_b_, CNN with M_4_, and DAN with M_4_ models. The comparison is based on various metrics, including the model size in terms of weights, computational cost in FLOPs, and inference time in seconds, etc.

During the inference stage, we observe that the number of weights in the multi-domain CNN-trained and multi-domain DAN-trained models is nearly double that of the CNN benchmark model. Consequently, this results in a notable increase in the inference time for multi-domain models. Thus, the inclusion of multi-domain CSI feature sets has enhanced positioning performance but at the cost of significantly increased model weights and inference time.

Among the multi-domain trained models, the DAN approach demonstrates a slight reduction in the number of weight parameters compared to the CNN method. However, it exhibits notably higher inference time due to the additional computational load introduced by the attention mechanisms. While pure CNN-based architectures are inherently more parallelizable on GPU hardware, the additive attention layers in DAN involve sequential operations and matrix multiplications that limit parallel efficiency. As a result, although the multi-domain DAN approach employs slightly fewer parameters, it incurs higher inference complexity than the CNN baseline, highlighting a performance-computational efficiency trade-off.

As shown in Table 3, the DAN with M_4_ achieves a modest accuracy gain over the CNN with M_4_, but its inference time nearly doubles (31.17 → 57.2 s for 50k samples). This overhead stems from the reduced parallelism of additive attention layers compared to convolutional operations. Nevertheless, the DAN architecture delivers more stable and reliable position estimates across varying data conditions, a property critical for sub-10 cm indoor positioning scenarios where robustness may often outweigh latency increments. For latency-sensitive real-time applications, CNN frameworks with multi-domain inputs may provide a better trade-off between precision and efficiency. Future work will explore transformer-based designs emphasizing dot-product attention to preserve DAN’s accuracy advantages while improving computational efficiency.

**Fig. 5:**
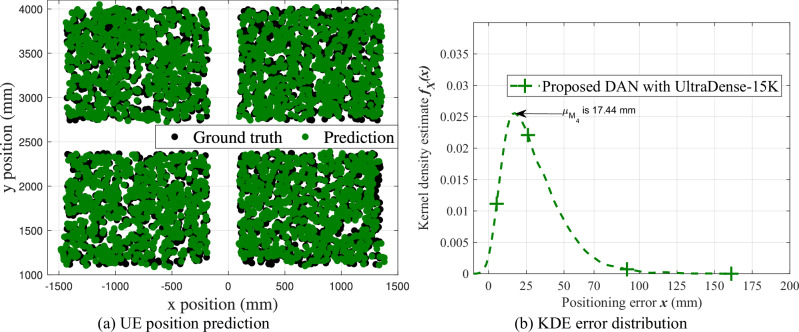
UE position prediction performance of the proposed multi-domain DAN model with M4 feature set trained using the UltraDense-15K dataset. **a** Comparison of 2D UE position predictions against ground truth from the UltraDense-15K dataset, where positions are predicted using the multi-domain DAN model trained with the M_4_ feature set. **b** KDE error distribution between predictions and ground truth for the proposed DAN model with the M_4_ feature set over the UltraDense-15K dataset.

Figure 5a illustrates the position predictions of the proposed DAN approach using the M_4_ feature set on the UltraDense-15K dataset, compared against the UE ground truth 2D positions. The results show that the DAN approach, trained with the M_4_ feature set, achieves position predictions closely matching their respective ground truths. This observation is further supported by Fig. 5b, which presents the corresponding KDE of the positioning errors. The mode error for the proposed DAN model with M_4_ is approximately 17.44mm across all UltraDense-15K CFR samples, indicating the high accuracy and consistency of the predicted positions relative to the ground truth. Overall, the proposed DAN approach with multi-domain features delivers improved positioning performance at the expense of increased inference complexity.

## Discussion

We proposed a multi-domain DAN framework to perform CSI-based UE indoor positioning using mMIMO wireless systems. We first constructed the multi-domain feature sets by extracting CSI-information from CFR, CIR, and CAR domains. We analyzed the position estimation performance using multi-domain feature sets over the CNN baseline and proposed DAN architectures. When training the CNN with multi-domain feature sets, it was observed that incorporating CAR domain features, in addition to CIR and CFR domain features, yielded better positioning. Applying this knowledge to attention mechanisms, we then compared the proposed DAN-based position prediction performance against the multi-domain CNN-baseline and previous CNN benchmark results. Our results show that the proposed multi-domain DAN approach favors superior position estimation performance than the CNN benchmark performance and the multi-domain CNN. Our analysis was extended on the DAN framework under different antenna configurations, a large number of CSI samples, number of training samples, and found that our approach offers stable and reliable performance. However, we also observe that incorporating these multi-domain features increases the model inference complexity, highlighting a performance-complexity trade-off. Despite this, our findings underscore the effectiveness of multi-domain CSI features, particularly from the CAR domain, and attention mechanisms in improving indoor UE positioning accuracy. For future research directions, we aim to investigate the inference complexity of complex multi-domain attention models like transformers, on GPU platforms to better understand their real-time deployment feasibility. Additionally, we plan to evaluate the generalization performance of both multi-domain CNN and DAN models in unseen indoor environments. Another important direction involves the creation of a comprehensive multi-domain CSI dataset tailored for complex and dynamic indoor scenarios, which will further facilitate research in robust indoor UE positioning.

## Methods

This section outlines features extracted from CFR data across multiple domains, including delay and angular domains, by applying Fourier transforms to derive the respective channel characteristics. Under each domain, we also construct features in both Cartesian and Polar representations to improve feature diversity and discriminability for the real-valued NN. These domain-specific features are then combined to form various uni-domain and multi-domain feature sets that comprehensively represent the wireless channel characteristics. These feature sets are used as inputs for learning frameworks to accurately predict UE positions. Finally, a complexity analysis of individual features and feature sets is presented. NN are powerful universal function approximators, capable of identifying complex mappings between multimodal data representations. Function approximators, such as fully connected networks, CNN, attention networks, etc. can learn highly non-linear relationships between inputs and outputs, making them suitable for tasks, such as CSI-based positioning. Fully connected networks, alternatively known as multilayered perceptrons, are suitable for learning direct, non-sequential mappings. CNN are primarily used for spatial or grid-like data, as they excel at capturing local patterns and hierarchies in input data, making them suitable for tasks, such as image-based positioning or spatial feature extraction from CSI data. Attention networks focus on learning the relative importance of different input features, allowing the model to attend to the most relevant parts of the input data dynamically. This is especially useful when dealing with temporal or spatial relationships where certain features might carry more weight than others.

We first investigate the usage of CNN architecture as a baseline [12] for UE positioning learning task using CSI feature sets. CNN are effective in capturing spatial dependencies within the CSI data using multi-domain feature sets. Subsequently, we examine the DAN architecture for UE positioning using CSI feature sets by integrating attention mechanisms into the CNN baseline. The attention mechanism enables the network to focus on the most relevant features within each domain and across different layers, enhancing its ability to learn complex domain-specific interactions and improve overall CSI-based positioning performance.

### Indoor mMIMO CSI Dataset Description

**Fig. 6:**
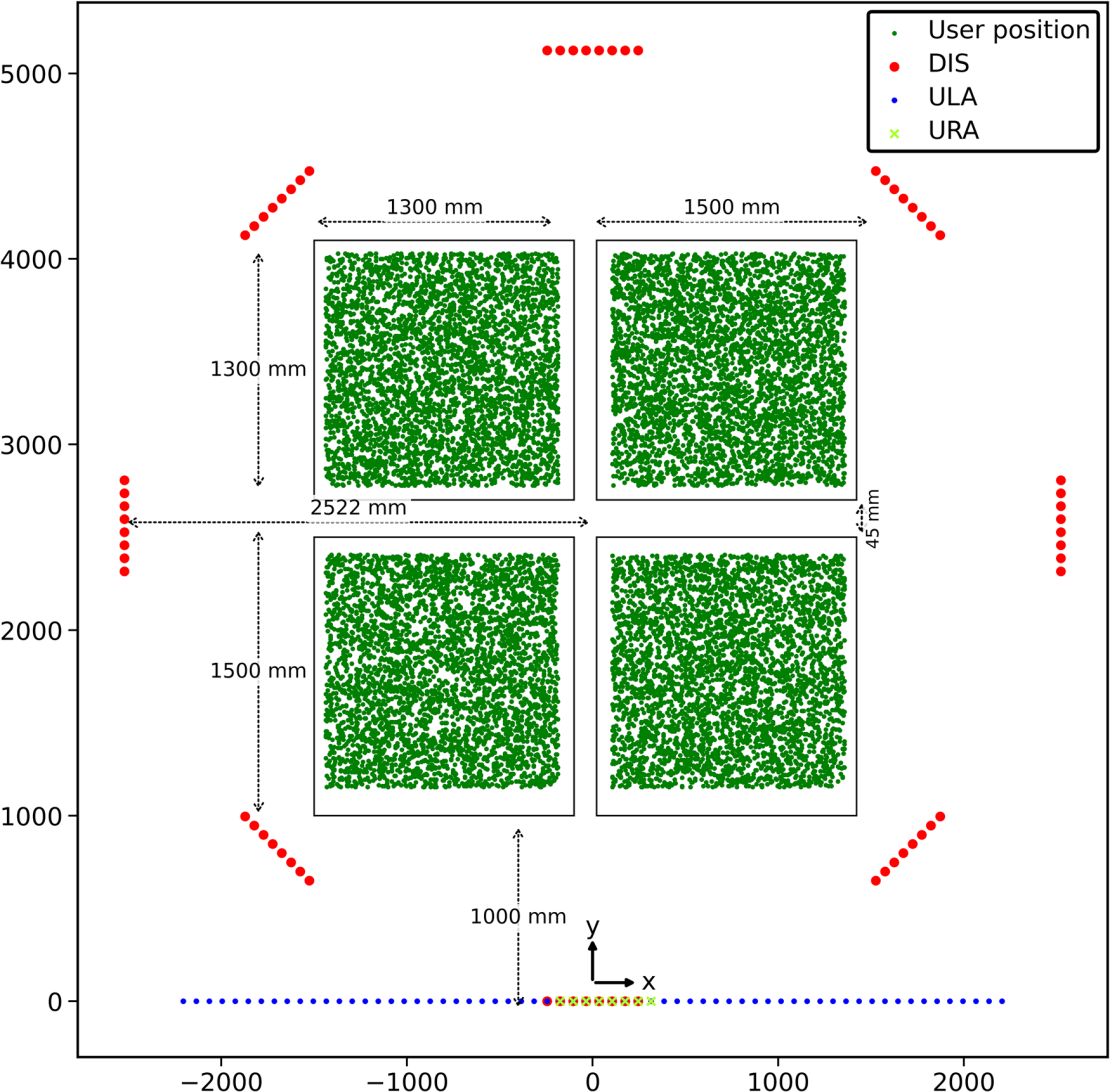
Ultra-dense mMIMO CFR dataset testbed setup [24]. Points inside the setup represent the positioning coordinates of the 15,000 samples in the dataset. The antenna configurations for ULA, URA, and DIS setups are shown in different colors.

We use the ultra-dense indoor CSI dataset^1^ measured by KU Leuven mMIMO testbed for many user positions [24]. In this context, CSI denotes frequency-domain measurements, corresponding to the CFR. The testbed consists of a BS with a mMIMO antenna system containing 64 antennas and the four universal serial radio peripherals (USRPs) with a single dipole antenna each, deployed within 3m × 3m target area as shown in Fig. 6. The BS acts as RX, receiving a pre-defined pilot signal from each UE following uplink communication. The pilot tone consists of 100 sub-carriers evenly spaced in frequency. For a single transmission, the measured CFR can be represented by the complex matrix,

1 $${{\bf{H}}}_{{\rm{CFR}}}=\{{H}_{{n}_{r},{n}_{k}}\}\in {{\mathbb{C}}}^{64\times 100},$$

where *n*_*r*_ = {1, 2, . . . . , 64} and *n*_*k*_ = {1, 2, . . . , 100} represent the antenna and sub-carrier indices, respectively. A center frequency of 2.61 GHz is used in the mMIMO testbed with a total bandwidth of 20 MHz and sub-carrier frequency spacing as 200 kHz. The transmitter power used is 15 dBm. The spacing between adjacent antenna elements was 70 mm and for the lowest antenna elements we located 93 cm above the floor. The user’s antenna was placed 20 cm above the floor. Using this setup, the area was scanned with 5 mm intervals, resulting in a dataset containing 252004 CFR samples with location accuracy of less than 1 mm. Besides, the BS testbed setup supports three different antenna array configurations, namely, ULA, URA and DIS. The URA setup employed an 8 × 8 antenna array and supported both LoS and nonline-of-sight (nLoS) scenarios. The ULA configuration featured a single line of 64 antennas while in DIS setup, the antennas were distributed over the room in pairs of eight, to achieve the distributed antenna deployment.

We define two datasets for the study, namely, *UltraDense-15K* and *UltraDense-50K*. UltraDense-15K dataset comprises 15,000 CFR samples and their corresponding UE ground truth 2D positions. This dataset is derived under ULA antenna configuration and obtained from the COST Interact 2020 ML Competition (https://codalab.lisn.upsaclay.fr/competitions/15314?secret_key=99b0bca3-705c-4a5f-bdfc-9c2d06a36718). We randomly split the dataset into 80% training and 20% testing and refer them as UltraDense-15K training and UltraDense-15K test datasets, respectively. UltraDense-50K dataset contains 50,000 CFR samples with their associated 2D ground truth UE positions. This dataset was constructed using a larger version of the ultradense dataset, featuring all three antenna configurations (ULA, URA, and DIS), as made available on the IEEE DataPort (https://ieee-dataport.org/open-access/ultra-dense-indoor-mamimo-csi-dataset). We prepare this dataset by initially selecting every fifth sample of 252,004 original samples. We perform this sampling to ensure and capture the entire range of UE positions under the sampled dataset. Next, we randomize the sampled dataset and randomly select 50,000 samples and address them as UltraDense-50K dataset. Finally, we split this dataset into 80% training and 20% testing and refer them as UltraDense-50K training and UltraDense-50K test datasets, respectively.

### Multi-domain CSI Feature Set Construction

In general, the positioning task utilizes channel data, i.e., CSI, which is inherently a time-series signal. CFR data obtained from (1) contains 2D channel information corresponding to 64 spatially distributed antenna elements and 100 sub-carrier frequencies. This 2D complex-valued information in Cartesian form is denoted as **f**_1_. The complex-valued elements in this subset can further be transformed into their polar representation to generate an additional feature subset, denoted as **f**_2_.

The time-series CFR data can be transformed into other domains by applying Fourier and Inverse Fourier transformations to its 2D channel representation. Specifically, we perform an Inverse Fourier transformation ($G(s,\tau)={{\mathcal{F}}}_{f}^{-1}({\bf{H}}_{\mathrm{CFR}})$) on the 2D CFR data along the frequency dimension (*n*_*k*_) to obtain the 2D delay-space domain CIR representation. This representation captures the channel characteristics across spatial and delay dimensions, consisting of complex-valued numbers in Cartesian form, and is denoted as the feature subset **f**_3_. We aim to construct new feature subsets to enhance further position prediction learning by providing more useful feature representations. Hence, the complex-valued numbers in this subset can be converted to polar form to generate an additional feature subset denoted as **f**_4_. In contrast to [12], which considered CFR and polar CIR, we further study the angular-frequency domain, where Fourier transforms expose not only frequency content but also beamspace (spatial-spectrum) characteristics. Such information may include the channel energy distribution across angles of arrival for each sub-carrier frequency, acting like a location fingerprint. We obtain the channel information in the angular domain by performing a Fourier transformation on the 2D CFR data ($B(f,\nu )={{\mathcal{F}}}_{s}({\bf{H}}_{\mathrm{CFR}})$) along the antenna index (*n*_*r*_) dimension. We define this 2D representation as the CAR domain. Similar to the previous feature subsets, the 2D CAR data can be represented in Cartesian and polar forms as **f**_5_ and **f**_6_, respectively. These feature subsets assist the learning framework in capturing beamspace characteristics across multiple subcarrier frequencies, thereby modeling the channel energy distribution corresponding to a UE’s spatial location relative to the BS RX.

**Fig. 7:**
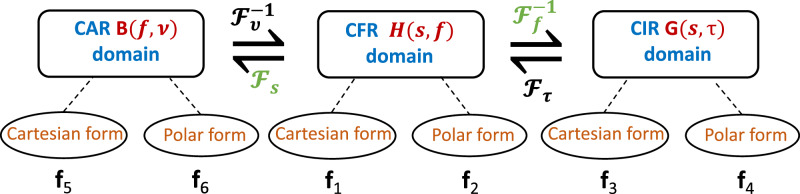
Multi-domain feature subset construction from CFR data (H(*s*, *f*) = H_CFR_) using Fourier and Inverse Fourier transformations. The CIR representation is derived via an inverse Fourier transform along the frequency domain ($G(s,\tau )={{\mathcal{F}}}_{f}^{-1}({{\bf{H}}}_{{\rm{CFR}}})$), while the CAR representation is obtained via a Fourier transform along the spatial (antenna) domain ($B(f,\nu )={{\mathcal{F}}}_{s}({{\bf{H}}}_{{\rm{CFR}}})$). For each domain, both Cartesian and polar representations are extracted to enrich feature diversity and enhance discriminability for real-valued neural networks.

Extracting features from multiple domains not only provides a deeper understanding of the signal behavior but also facilitates the identification of underlying spatial, temporal, and spectral patterns that can significantly enhance positioning performance. Consequently, multi-domain features offer a more comprehensive and discriminative representation compared to single-domain data. The CSI-based position prediction study in [12] is performed by combining information from **f**_1_, **f**_2_, and **f**_3_ feature subsets. Overall, we construct six feature subsets from three domains namely, CFR, CIR, and CAR. We hypothesize that a multi-domain approach enhances position learning performance and improves its predictive accuracy by providing a richer and more detailed set of features. The features subsets constructed by Fourier transformations are illustrated in Fig. 7 and can be summarized as follows:

- **f**_1_:7D2*Cartesian coordinates of the CFR data*, i.e. **H**_CFR_,
- **f**_2_:7D2*Polar coordinates of the CFR information*, i.e. ${\mathcal{P}}\left({{\bf{H}}}_{{\rm{CFR}}}\right)$,
- **f**_3_:7D2*Cartesian coordinates of the CFR information*, i.e. ${{\mathcal{F}}}_{f}^{-1}({{\bf{H}}}_{{\rm{CIR}}})$,
- **f**_4_:7D2*Polar coordinates of the CFR information*, i.e. ${\mathcal{P}}({{\mathcal{F}}}_{f}^{-1}({{\bf{H}}}_{{\rm{CFR}}}))$,
- **f**_5_:7D2*Cartesian coordinates of the CAR information*, i.e. ${{\mathcal{F}}}_{s}({{\bf{H}}}_{{\rm{CFR}}})$,
- **f**_6_:7D2*Polar coordinates of the CAR information*, i.e. ${\mathcal{P}}\left({{\mathcal{F}}}_{s}({{\bf{H}}}_{{\rm{CFR}}})\right)$,

where ${\mathcal{P}}$, ${{\mathcal{F}}}^{-1}$ and ${\mathcal{F}}$ imply polar form representation, inverse fast Fourier transformIFFT and fast Fourier transformFFT respectively. Table 4 details the combinations of the features used to construct different feature sets. U_1_, U_2_, U_3_ corresponds to the sets constructed only from uni-domain features namely, CFR, CIR, and CAR, respectively. Similarly, M_1_, M_2_, M_3_, and M_4_, correspond to the sets constructed from multiple domain features. M_*b*_ is the multi-domain feature set used in [12].

### Multi-domain Feature Set Complexity Analysis

In this work, the complexity of building a set of features is evaluated by the number FLOPs. The feature subset **f**_1_ can be readily extracted from the CSI matrix in (1). Construction of feature subset **f**_2_ involves converting the values from cartesian to polar conversion in **f**_1_. Computation of the magnitude of a complex number involves two squaring operations, one addition, and one square root operation which require two FLOPs, one FLOP, and one FLOPs respectively [27]. Similarly, computation of the argument of a complex number involves one division and an arctangent operation, which require one FLOP each. For each data sample, the computation of magnitude and argument requires 4 and 2 FLOPs respectively. Therefore, the complexity to construct **f**_2_ with *N* subcarriers and *N*_*t*_ transmitter antennas is

2 $${{\mathcal{C}}}_{({{\bf{f}}}_{2})}=6\,N\,{N}_{t}.$$

The construction of feature subset **f**_3_ involves an IDFT operation. By using FFT algorithm, we require $6N{\log }_{2}N$ FLOPs per antenna. Therefore, the complexity to construct **f**_3_ with *N* subcarriers and *N*_*t*_ transmitter antennas is

3 $${{\mathcal{C}}}_{({{\bf{f}}}_{3})}=(6N {\log }_{2}N)\,{N}_{t}.$$

Construction of feature subset **f**_4_ involves converting the values from cartesian to polar conversion in **f**_3_. From (2), we can infer that the complexity ${{\mathcal{C}}}_{({{\bf{f}}}_{4})}$ and ${{\mathcal{C}}}_{({{\bf{f}}}_{6})}$ is 6*NN*_*t*_ FLOPs. Construction of feature subset **f**_5_ involves an discrete Fourier transform (DFT) operation and needs $6N{\log }_{2}N$. But, this can be further reduced if, during the IDFT operation, we can use the conjugate of complex exponential factor and then use it the same to perform DFT. As per Table. 5, the conjugation operation does not require any FLOPs [28]. Therefore, the construction of feature subset **f**_5_ doesn’t involve any additional complexity.

**Table 5:** Complexity of different mathematical operations in terms of the number of floating-point operations (FLOPs) [27, 28]

Operation	Remark	FLOPs count
*a* − *jb*	Conjugation of a complex number	0
*a* + *b*	Real addition	1
*a* − *b*	Real subtraction	1
*a* × *b*	Real multiplication	1
*a*/*b*	Real division	1
$\sqrt{a}$	Real square root	1
(*a* + *jb*) + (*c* + *jd*)	Complex addition	2
(*a* + *jb*) − (*c* + *jd*)	Complex subtraction	2
(*a* + *jb*) × *c*	Complex-real multiplication	2
$\arg (a+jb)$	Argument of a complex number	2^*a*^
∣*a* + *jb*∣	Magnitude of a complex number	4
(*a* + *jb*) × (*c* + *jd*)	Complex-complex multiplication	6

The table summarizes the computational cost of common real and complex arithmetic operations.

^*a*^A lookup-table approach is assumed for trigonometric computations (sine, cosine, and arctangent), where the search operation counts as one FLOP The number of FLOPs for these three operations may vary from 5 to 10 depending on implementation.

^*b*^A lookup-table approach is assumed for trigonometric computations, such as sine, cosine, and arctangent, where the search operation is counted as one FLOP.

### CNN Baseline architecture for Multi-Domain

Pollen et.al. in [12] highlights the importance of using CNN to process the CFR samples and infer their spatial information. They leverage convolutional layers to automatically and adaptively learn spatial hierarchies and structures of features from the input CFR data. Mathematically, the operation of a CNN layer can be written as:

4 $${\bf{y}}=f\left({\bf{W}}* {\bf{x}}+{\bf{b}}\right),$$

where **y** is the output feature map (post-activation), **W** is the convolutional kernel (filter), * represents the convolution operation, **x** is the input feature map, **b** is the bias term, added to each output feature map, *f*( ⋅ ) is the element-wise applied activation function. CNN are available in various configurations, such as 1D, 2D, or 3D, depending on the convolutional kernels’ dimensionality and the input data’s characteristics. The selection of a specific CNN type is guided by the data’s structure and the dimensions along which critical information needs to be extracted.

**Fig. 8:**
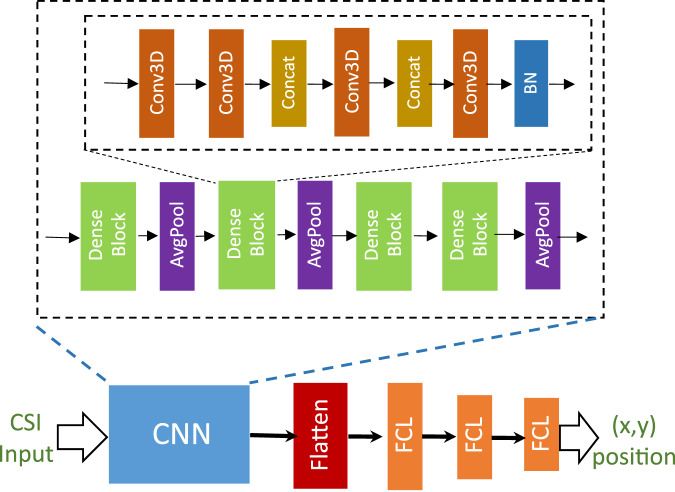
CNN baseline architecture used for the mMIMO CSI dataset [12]. This architecture is employed in this work to analyze both uni-domain (U1, U2, and U3) and multi-domain CSI-extracted (M1, M2, M3, M4, and Mb) feature sets.

Figure 8 presents the CNN baseline architecture proposed for the mMIMO CSI dataset [12]. We adopt the same CNN structure but extend it by incorporating our multi-domain CSI feature sets, stacking all constructed features along the third dimension of 3D CNN. In contrast to the CNN implementation in [12], we add an extra fully connected dense layer to effectively accommodate the increased feature dimensions. Mathematically, the high level for the predicted output ($\hat{y}$) from CNN baseline architecture can be written as

5 $${\hat{y}}_{{\rm{CNN}}}=\mathrm{FC}\,\left(\,\mathrm{CNN}\,(X)\right),$$

where $X=\,\mathrm{Concatenate}\,\left({X}_{{\rm{CIR}}},{X}_{{\rm{CFR}}},{X}_{{\rm{CAR}}}\right)$ are the concatenated multi-domain CSI input, CNN denote the convolutional block, and FC denote the fully connected layer as shown in Fig. 8. For a training batch with *n* samples, we implement the mean squared error (MSE) loss function (${{\mathcal{L}}}_{{\rm{CNN}}}$) between ground truth 2D UE position (*y*) and predicted output from CNN baseline architecture (${\hat{y}}_{{\rm{CNN}}}$) as follows:

6 $${{\mathcal{L}}}_{{\rm{CNN}}}=\frac{1}{n}| | {\hat{y}}_{{\rm{CNN}}}-y| {| }_{2}^{2}.$$

Thus, the multi-domain CNN baseline model processes data along all dimensions, enabling it to effectively learn CSI-extracted feature information and identify relevant patterns for indoor UE positioning tasks.

### Multi-domain Deep Attention Network

Attention networks are popularly used in natural language processing applications like ChatGPT and also in computer vision tasks to address the spatial nature of image data alongside CNN [29]. Attention mechanisms allow models to recognize and focus on the most relevant parts of the input data. In our implementation, we have used attention mechanisms between layers, which correspond to the extracted abstract features from the given CSI input data passed through 3D CNN layers. We hypothesize that adding attention mechanisms between the hidden layers of 3D CNN deep networks helps the model to focus on the important components from the extracted features. The Q, K, and V are the core components of the attention mechanisms. Conceptually, Q represents the element that is evaluated, K is used to determine the relevance of other elements, and V contains the weighted and aggregated information based on the relevance determined by Q and K. In our implementation, as we take the attention values between two layers *l*_1_ and *l*_2_ for instance, the Q is *l*_1_ and the K and V are both *l*_2_. The attention weights are computed by comparing Q and K, using either an additive or a dot-product method, and these weights are then used to calculate a weighted sum of V, producing a context vector as the output of this attention mechanism. When an attention network integrates into the CNN architecture, this context vector enables dynamic feature refinement by focusing on the most relevant parts of the CSI data, ultimately improving the model’s ability to capture contextual information for more accurate UE positioning.

**Fig. 9:**
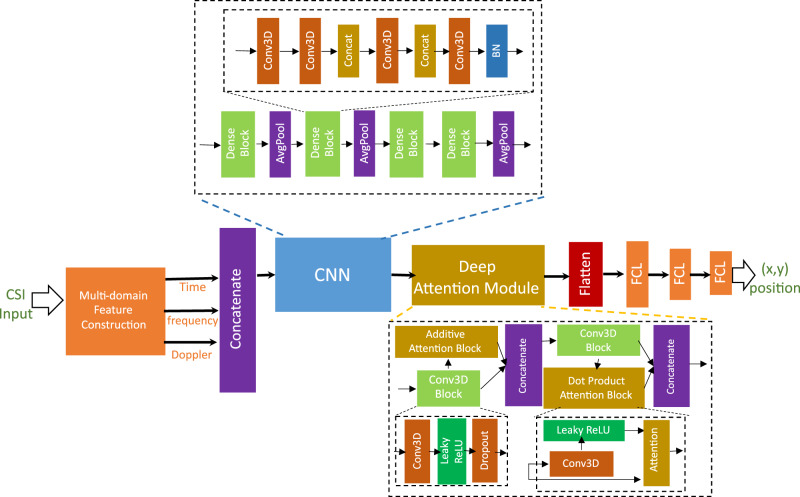
Proposed multi-domain DAN framework for indoor CSI-based positioning. This architecture is evaluated across multiple multi-domain feature sets using the mMIMO CSI dataset, demonstrating the effectiveness of attention-based feature fusion for accurate position estimation.

The end-to-end architecture of the proposed DAN framework for CSI-based positioning is shown in Fig. 9. We adopt the same CNN architecture described in Section 4.4 and incorporate a deep attention module between the CNN layers and fully connected layers of the baseline architecture. For the attention module, we use a combination of two prominent attention mechanisms namely, *additive attention*[30] and *dot product attention*[31]. *Additive attention* calculates attention scores from the hyperbolic tangent over a sum of Q and K, which are then normalized using a softmax function. Similarly, *dot product attention* calculates attention scores by taking the dot product of query and key vectors [32], measuring their similarity. The attention score for the additive attention or a dot product attention operation can be formulated as

7 $${\alpha }_{ij}=\left\{\begin{array}{ll}\,\mathrm{softmax}\,(\tanh \left({{\bf{Q}}}_{i}+{{\bf{K}}}_{j}\right))\qquad \mathrm{if}\,{additiveattention}\\ \;\mathrm{softmax}\,\left(\frac{{{\bf{Q}}}_{i}^{\top }{{\bf{K}}}_{j}}{\sqrt{{d}_{K}}}\right)\qquad\quad\qquad\;\mathrm{if}\,{dotproductattention}\end{array}\right.,$$

where *α*_*ij*_ is the attention weight (or alignment score) between the Query **Q**_*i*_ and the Key **K**_*j*_ vectors, *i*,*j* are the neural network layer indices, $\tanh (\cdot )$ is the activation function applied to the sum of the Query and Key, ${d}_{{K}_{j}}$ is the dimension of the *K*_*j*_ key vector, and $\sqrt{{d}_{{K}_{i}}}$ is the scaling factor to prevent large values of the dot product, softmax( ⋅ ) normalizes the attention scores to ensure they sum to 1. The attention mechanism output (also known as the context vector) **C**_*j*_ is then computed as a weighted sum of the Value (V) vectors, such as **C**_*j*_ = ∑_*i*_*α*_*ij*_**V**_*i*_, where **C**_*j*_ is the context vector at network layer *j*, *α*_*ij*_ is the attention weights between *i*, *j* network layers, that determines how much each Value (V) vector contributes to the context vector **C**_*j*_. This attention mechanism output is then concatenated to the extracted features from the Conv3D block inside the deep attention module architecture. We observed that our deep attention module with a combination of additive and dot product attention mechanisms is the best for handling the multi-domain CSI input. This approach aims to enhance CNN’s ability to capture complex features from different levels of abstracted features, and improve the accuracy of UE positioning estimation. Mathematically, the high level form of the predicted output ($\hat{y}$) from the DAN can be written as follows:

8 $${\hat{y}}_{{\rm{DAN}}}=\mathrm{FC}\,\underbrace{\left(\,\mathrm{Conv3D}\,\left({\mathrm{Attention}}_{{\rm{dot}}}\left(\,\mathrm{Conv3D}\,\left({\mathrm{Attention}}_{{\rm{add}}}\right)\right)\right)\right)}_{{\mathrm{deep}}\, {\mathrm{attention}}\, {\rm{module}}}\left(\,\mathrm{CNN}\,(X)\right),$$

where $X=\,\mathrm{Concatenate}\,\left({X}_{{\rm{CIR}}},{X}_{{\rm{CFR}}},{X}_{{\rm{CAR}}}\right)$ denote the concatenated multi-domain CSI input, CNN denotes the convolution block, Attention_dot_, Attention_add_ denotes the dot product and additive attention blocks, Conv3D denotes the 3D convolutional operation block inside deep attention module, and FC denotes the fully-connected layer block as shown in Fig. 9. For a training batch of *n* samples, we implement a MSE loss function (${{\mathcal{L}}}_{{\rm{DAN}}}$) between ground truth 2D UE position (*y*) and predicted output from multi-domain deep attention network architecture (${\hat{y}}_{{\rm{DAN}}}$) as

9 $${{\mathcal{L}}}_{{\rm{DAN}}}=\frac{1}{n}| | {\hat{y}}_{{\rm{DAN}}}-y| {| }_{2}^{2}.$$

Thus, the multi-domain DAN architecture aims to focus on the most relevant parts of the CSI data and helps to estimate UE positioning more accurately.

## Data Availability

No datasets were generated or analyzed during the current study. The datasets analyzed in this study are derived from COST Interact 2020 ML competition (https://codalab.lisn.upsaclay.fr/competitions/15314?secret_key=99b0bca3-705c-4a5f-bdfc-9c2d06a36718) and IEEE Dataport (https://ieee-dataport.org/open-access/ultra-dense-indoor-mamimo-csi-dataset).
